# On Some Points in the Treatment of Diabetes—II

**Published:** 1891-09-12

**Authors:** 


### THE PRACTITIONER'S MIRROR AND RETROSPECT.

[*The Editor will be glad to receive offers of co-operation and contributions from members of the profession. All letters should be addressed to* THE EDITOR, THE LODGE, PORCHESTER SQUARE, LONDON, W.]

### MEDICINE.

### ON SOME POINTS IN THE TREATMENT OF DIABETES.—II.

In the gouty cases if the sugar is reduced to a small amount and the lithiasis carefully treated, all distressing symptoms may disappear and life may go on to eighty or ninety years. Professor Naunyn lays down that levulose, enosit, inulin, and mannit, *i.e.*, the sugars which produce left-handed polarization, are eaten by diabetics without harm, while the right-sided sugars are poisonous.

If we turn to actual therapeutics we find ourselves confronted with a vast number of rejected drugs, almost every new therapeutic agent being tried as it comes out, praised by some illogical observer for effects it never produced, and then thrown aside. The only drugs of generally recognised importance are opium and its derivative codeia. The sugar, which has been lessened by diet, can be still more reduced by by these if given increasing doses, and in gouty cases the alkaline waters form an indispensable part of the treatment. Saundby, indeed, states that alkalies and salicylate of soda do not diminish the amount of sugar, yet insists strongly on their utility. His remarks on the risk caused to diabetics by the shock and fatigue of long railway journeys, and his advice to try the Carlsbad and other waters at home instead of travelling to the springs, appear to be dictated by sound common sense.

Jambul, antipyrin, ouabain have met with modified success in mild cases, and the adherents of the pancreatic theory of diabetes have tried some form of zymine. Could we be sure of the existence of pancreatic failure in any given case, it would be desirable to try the effect of the transplantation of a piece of healthy pancreas, as recommended in the case of thyroid disease.

With the regulation of the diet, the administration of opium, and alkaline waters, our powers of direct medicinal treatment come almost to an end.

Bathing and massage do often afford much relief, and it is almost unnecessary to refer to the enormous importance of protecting our patients from cold, fatigue, and mental worry. Any sudden shock from either cause, even any slight illness such as influenza, may be followed by rapid collapse, which is relieved by no remedy, and ends rapidly in death.

In considering the complications which we meet with, the question at once arises whether gout and the troubles produced by uric acid are secondary to the chronic form of diabetes in later life, or the actual cause of it, or, at any rate, symptoms of the pathological state which gives rise to it. We have already touched upon the consideration how far a diabetic diet should be rigorously maintained in the presence of lithiasis. There seems reason to decide that it is desirable to employ at first a strict diet for a short time in these cases to get rid of the irritation produced by the passage of sugar through the urinary organs, and to complete, if necessary, this purification by the use of opium or codeia. Afterwards the gouty state must be carefully treated, and probably the diet may have to be modified with this view. Indeed, from the first it is important to regulate the quantity, lest we cause a hypersecretion of uric acid. Hence the need of frequently testing both the water and the weight of the body, that we may discover when and how far a rigid diet requires to be modified. When we have thus reduced the sugar to a minimum the administration of salicylates for a considerable period may aid in preventing a relapse of the gout, and an occasional return to strict feeding may suffice to keep in check the diabetes.

The entire body is in a state of imperfect nutrition, and hence failure of any important organ may easily occur, but the processes of repair are difficult. How this malnutrition is brought about is not easy to explain. It can hardly be the irritation due to the presence of sugar in excess, for, as Saundby points out, any great surplus of sugar in the blood is quickly eliminated by the kidneys; nor is it clear that the constant drain of the excretion is the active cause. Flannel under-garments are important on account of the liability to rheumatic affections and to chest complications. The latter, indeed, often take a rapid and fatal course. Thus, in the influenza epidemic of 1890, amongst 300 cases treated by the writer, there were two patients who had suffered from a mild form of diabetes. One, indeed, recovered after a prolonged bronchial attack, but the other, who was in active work up to the attack of influenza, succumbed at once in a remarkable manner with symptoms of profound collapse when the lungs became affected.

It has been debated whether diabetic patients are liable to phthisis. Addison and Wilks considered that what appears to be phthisis in them, and is frequently fatal, is really a chronic pneumonia. Fagge, too, thought that the affection was usually not tubercular in origin; but some of the cases have shown undoubted evidence in favour of the other view. However, the majority appear to be instances of a chronic pneumonia, which quickly produces destructive changes and goes on to a fatal termination. Passing over the liability to gangrene, perforating ulcer, and frequent toothache, we come to the most serious complication of the disease, diabetic coma in either of its three types. It may commence with symptoms of collapse, of intoxication, or of dyspnœa. Sometimes it follows a slight fatigue, or an attack of constipation, to which diabetics are very subject. It has been thought to be induced at times by a strict meat diet, but Fagge and Saundby point out that it occurs also on ordinary diet. Mental excitement, fatigue, or any severe pain are probably the most usual antecedents. Unfortunately, we know very little which can be definitely proved, either as to the pathology or the proper remedies for this state. It has been variously ascribed to the presence of acetone, aceto-acetic acid, oxybutyric, or an excess of other acids in the blood, lipœmia and heart failure, but no certain results have been attained. The earliest symptoms are usually "deep respiration, rapid pulse, and abdominal pain," and not unfrequently a peculiar scent like apples or hay.

Saundby, whose discussion of the pathology is, perhaps, the most complete, lays stress on the occurrence of epigastric pain, followed by feebleness and rapidity of the pulse, and quickened breathing, with prolonged inspiration and expiration. Cyanosis, and sometimes convulsions, only appear just before death, but drowsiness is found at an earlier stage. As to an examination of the water, all that can be said is this, perchloride of iron sometimes produces with urine a reddish-brown or Burgundy red colour. If this disappears on heating in the case of a diabetic patient there is considerable probability of an attack of coma coming on. We have at present no more certain test than this, nor is it possible to say what is the substance which causes the reaction. Saundby holds that little diagnostic importance can be attached to the presence of acetone as shown by the gradual appearance of a rose-violet colour when the nitro-prusside of sodium and ammonium is added to urine. However, the ferric-chloride reaction has a practical value, and should always lead to precautionary measures, especially to free purgation and rest in bed. Putting on one side the cases where collapse and coma, or the peculiar scent and coma are the symptoms, we find in typical cases a restless excited state, exaggerated respiration, a weak rapid pulse, with falling temperature, and finally coma, from which an almost invariably fatal result follows. Stadelman, who like Minkowski, holds this condition to be due to an excess of oxybutyric acid, recommends large doses of an alkali, or tartrate of soda, to be given by the mouth as early as possible, since he confesses that previous attempts by the injection of alkalies have only resulted in failure. These injections of 3 or 4 per cent. sodium carbonate in a solution of sodium chloride have been tried by Hesse, Dickenson, Von Ziemsen, and others, from the time when coma was first described. Occasionally temporary improvement has resulted, but no permanent good has followed.

Ralfe recommends a vapour bath in bed, with doses of ether, ammonia, and musk. Jaccoud, in addition to the administration of alkalies, would purge and give inhalations of oxygen. That the acidity of the blood in some of these cases is very great, appears from the impossibility at times of rendering the urine alkaline with the large doses of soda that have been given. The most reasonable plan of treatment seems to be a sharp purge, followed by stimulants, and disinfectants for the intestinal tract, with large doses of alkalies if time admits. Until we know what is the poison, there is little use in doing anything else than supporting the heart by stimulants. The only indication for treatment yet known is the acid state of the blood, and, as we have said, attempts to treat this condition have hitherto failed, though we are of opinion that they should still be employed as early and as vigorously as possible.

